# Supplementary material for: Acceptance and compliance with micronutrient powder (MNP) among children aged 6–23 months in northern Nigeria
Source: PLOS Glob Public Health. 2022 Oct 17;2(10):e0000961. doi: 10.1371/journal.pgph.0000961 (PMC10022258; doi:10.1371/journal.pgph.0000961)
Supplement: S1 Dataset — (ZIP) [file pgph.0000961.s008.zip › S8_Dataset/Direct Observations.zip!/Direct Observations/28605_A_GU_AD_1_DO.pdf]

1.1.1 Instrument F –Direct Observation Form

**<<Instrument F Direct Observation Form>>**
**PI Name:** PragyaMathema,Stephen Kodish<br>**Study Title:** Formative research to develop an infant and young child feeding (IYCF) intervention with micronutrient powder (MNP) for children aged 6 – 23 months<br>**PI Version/Date:** Version1.0/January 24, 2015

Date: _July 01, 2015____________Location: _Bongo community, LGA -Song

Data Collector: Aisha Aliyu Dahiru_______ Participant ID: __

Main goals of this direct observation are to answer the following questions:

1. In this household, what would be the best way(s) to give nutritional supplements to young children? Why?

2. In this household, describe the food sharing that you observe throughout the meal. Are there any predictable patterns?

3. In this household, who is favored and how is this favoritism expressed during meal preparation and eating?

(TEAR OFF AT PERFORATED DOTTED LINE BELOW)

PARTICIPANT ID: __

Household Composition Table (Fill out as you do data collection)

INITIALS	SEX (M/F)	AGE (estimated)	Relationship to household	Description
M	F	24YRS	Mother	Wearing purple dress.
B	M	11 MONTHS	Baby boy (son)	Wearing green trouser and shirt
N1	F	24 YRS	Neighbor	Wearing brown head gear with blue wrapper
H	M	32 YRS	Husband	Wearing white shirt with black trouser
N2	M	34 YRS	Husband's friend	wearing white shirt black trouser
N3	F	32 YRS	Husband friend	Wearing brown dress

PARTICIPANT ID: __________

TIME	ACTOR INITIALS	RECIPIENT INITIALS	WHAT HAPPENED	FOOD	Reactivity
8.00	M		Sweeping		
8:05	M		Sweeping		
8:10	M		Sweeping		
8:15	M		Washing hands		
8:20	M		Washing utensils		
8:25	H	B	Playing with baby sitting on a stool		
8:30	M	H	Serving food to the husband	Tuwon dawa, miyan kuka	
8:30	H		Husband washing his hands		
8:30	M	OC,OM	Serving food to older son and his cousin	Tuwon dawa, miyan kuka	
8:31	M		Washing her hands		
8:31	H	B	Husband feeding the child sitting on the ground	Tuwon dawa, miyan kuka	

TIME	ACTOR INITIALS	RECIPIENT INITIALS	WHAT HAPPENED	FOOD	Reactivity
8:32	M	B	Mother feeding the baby from a separate bowl	Tuwon dawa , miyan kuka	
8:33	OC		Eating from the same bowl with his cousin on the side	Tuwon dawa, miyan kuka	
8:34	M	B	Mother assisting the child to eat from her bowl	Tuwon dawa, miyan kuka	
8:35	M	B	Giving child water to drink	Water	
8:36	N		Comes in chatting with mother		
8:37	N		Drink water from the child cup	Water	
8:37	M	N	Chatting with neighbor		
8:37	H	N	Chatting with neighbor		
8:38	M	B	Still assisting the child to eat	Tuwon dawa, miyan kuka	
8:40	H	OC	Washing his hands and hands the remaining food to the older child		
8:40	M	B	Washing the child hands and wiping his mouth		
8:45	H	B	Playing with baby on his laps		
8:45	M		Serving more food for herself and sits beside the husband and child eating	Tuwon dawa, miyan kuka	
8:50	OC		Eating with his cousin	Tuwon dawa, miyan kuka	
8:50	OM		Eating with his cousin	Tuwon dawa	
8:55	OM		Washing his hands		
8:55	OM		Drinking water from the clay pot under the shade	Water	
9:00	OC		Still eating alone	Tuwon dawa,	

TIME	ACTOR INITIALS	RECIPIENT INITIALS	WHAT HAPPENED	FOOD	Reactivity
				miyan kuka	
9:00	M		Eating beside the husband and child playing	Tuwon dawa, miyan kuka	
9:05	B		Sitting beside the mother playing		
9:10	B		Eating with his hands from the mother's bowl	Tuwon dawa, miyan kuka	
9:11	M	B	Assisting the child to eat	Tuwon dawa	
9:12	H	M,B	Chatting with the child and the mother while they are eating	Tuwon dawa, miyan kuka	
9:12	OC		Washing the utensils under a tree		
9:12	OM		Assisting the older child to wash the utensils		
9:13	M	B	Assisting the child to eat	Tuwon dawa, miyan kuka	
9:14	H	M,B	Chatting with mother and child while they are eating	Tuwon dawa	
9:15	H	B	Playing with the baby and washing his hands		
9:15	M		Eating alone	Tuwon dawa	
9:15	M	B	Giving the child water to drink from a cup	Water	
9:20	M		Washing her hands		
9:25	H	B	Playing with the baby sitting on a stool while the child is on the ground		
9:30	M	B	Carrying the child on her laps playing with him		

TIME	ACTOR INITIALS	RECIPIENT INITIALS	WHAT HAPPENED	FOOD	Reactivity
9:35	OC		Taking utensils washed into the shade		
9:40	OM		Going out together with the older child chatting		
9:40	M	B	Playing with the child on her laps		
9:45	H	M	Chatting with the mother		
9:45	B		Coughing so much		
9:50	M	B	Playing with the child on her laps		
9:55	B		Crying		
9:55	M	B	Consoling the child		
10:00	H	B	Playing with the child while the child is sitting on the mothers lap		
10:05	H	M	Chatting with the mother		
10:10	M	B	Playing with the child on her laps while chatting with the husband		
10:15	B		Playing on mother's lap		
10:20	H	M	Chatting with mother showing her something on his mobile phone		
10:25	N2		Comes in and sit on a stool beside the mother and the husband		
10:30	M	N2	Chatting with the neighbor		
10:35	H	N2	Chatting with the neighbor		
10:40	HB1		Enter the and bring out a bowl of food on a tray		
10:40	HB2		Washing his hands		
10:40	B	HB1,HB2	Serving husband's brothers drinking water in a bowl	water	R
10:45	HB1		Eating in the same bowl with brother	Tuwon dawa, miyan kuka	
10:45	HB2		Eating in same bowl with brother	Tuwon dawa, miyan	

TIME	ACTOR INITIALS	RECIPIENT INITIALS	WHAT HAPPENED	FOOD	Reactivity
				kuka	
10:50	B		Sitting beside uncle's playing on the ground		
10:55	M	HB1,HB2	Chatting with husband's brother while they are eating	Tuwon dawa. Miyan kuka	
11:00	B		Playing near the uncle while they are eating		
11:05	M		Goes into the room		
11:10	B		Still playing near the uncle, making motorcycle noises and touching the older uncle		
11:15	B		Sitting behind the younger uncle playing		
11:20	M	HB1,HB2	Chatting with husband brothers		
11:25	M	HB1,HB2	Chatting with husband brothers		
11:25	HB1		Still eating with brother		
11:25	HB2		Still eating with brother		
11:30	H	HB1,HB2	Chatting with brothers		
11:35	HB2		Washing hands		
11:35	HB2		Drinking water	Water	
11:40	HB1		Washing hands		
11:40	HB1		Drinking water	Water	
11:40	HB1		Drinking water	Water	
11:50	M	N3	Serving food to neighbor	Tuwon dawa	
11:55	M	N3	Serving water in a large cup to neighbor	Water	

TIME	ACTOR INITIALS	RECIPIENT INITIALS	WHAT HAPPENED	FOOD	Reactivity
11:55	B		Playing under the shade the alone		
12:00	M	N3	Mother chatting with neighbor while his eating	Tuwon dawa, miyan kuka	
12:05	B		Playing with a large plastic container		
12:10	B		Playing with a large plastic container		
12:10	M		Putting water from clay pot into a plastic bucket		
12:15	B		Playing near the bathroom door		
12:15	B		Crying		
12:20	M	N3	Chatting with neighbor while he is eating	Tuwon masara, miyan kuka	
12:20	B		Running towards the mother laughing and spreading his hands		
12:25	M	B	Carrying the child on her laps		
12:30	M	B	Breast feeding the child	Breast milk	
12:35	M	N3	Chatting with neighbor while his eating	Tuwon dawa, miyan kuka	
12:40	M	B	Breastfeeding the child	Breast milk	
12:35	M	N3	Chatting with neighbor while eating	Tuwon dawa, miyan kuka	
12:40	M	B	Breastfeeding the child	Breast milk	
12:45	M	N3	Chatting neighbor white he is	Tuwon	

TIME	ACTOR INITIALS	RECIPIENT INITIALS	WHAT HAPPENED	FOOD	Reactivity
			eating	dawa, miyan kuka	
12:50	B		Playing on mother's		
12:55	M	N3	Assisting the neighbor to wash hands while the baby watch her		
12:55	B		Playing beside mother and neighbor		
1:00	B		Playing with sand and water		
1:05	B		Eating the wet sand		
1:10	B		Rubbing the wet sand on bag		
1:10	M	B	Washing the child hands		
1:15	M		Bringing out mortar and pestle from the room		
1:15	M		Bringing out beans from the room	Beans	
1:15	M		Puts beans in mortar and add water	Beans	
1:20	M		Pounding beans to remove back	Beans	
1:25	B		Playing around the house		
1:30	H	B	Comes out from the room and stands playing with the child		
1:35	H	B	Playing with the child and walking towards the main entrance		
1:40	B		Crying		
1:40	B		Crying		
1:45	H	B	Carrying child to the mother, placing him on her lap		
1:45	M	B	Breastfeeding baby sitting on her lap	Breast milk	
1:50	M	B	Breast feeding child while pounding beans to remove skin	Beans	
1:55	M	B	Breastfeeding the child	Breast	

TIME	ACTOR INITIALS	RECIPIENT INITIALS	WHAT HAPPENED	FOOD	Reactivity
				milk	
1:55	M		Pounding beans to remove the back	Beans	
2:00	M		Pounding beans to remove the back	Beans	
2:00	B		Child playing with stick beside the mother		
2:05	B		Crying and scratching his head		
2:10	B		Crying		
2:10	B		Crying and comes to sit on my lap	R	
2:15	B		Quietly sitting on my lap	R	
2:20	M	B	Trying to carry child but he refuses	R	
2:25	B		Quietly sitting on lap	R	
2:30	B		Sitting on the ground crying and rubbing his eyes		
2:35	B		Child crying		
2:40	B		Child crying and screaming		
2:40	B		Sleeping while sitting		
2:40	M	B	Laying baby down on a wrapper under the shade		
2:45	B		Sleeping		
2:50	B		sleeping		
2:50	M		Still pounding beans to remove back		
2:55	B		Sleeping under the shade		
2:55	M		Pounding beans lightly to remove back		
3:00	M		Goes into room and bringing out drying mat		
3:05	M		Spreading beans on drying mat		
3:10	M	B	Checking on sleeping child		
3:15	B		Sleeping		
3:20	B		Sleeping		
3:25	B		Sleeping		

TIME	ACTOR INITIALS	RECIPIENT INITIALS	WHAT HAPPENED	FOOD	Reactivity
3:30	M		Sitting on stool under the shade		
3:35	M		Cutting nails and singing		
3:40	B		Sleeping		
3:45	M		Chatting with neighbor		
3:45	M	N1	Chatting with neighbor		
3:50	M	N1	Chatting with neighbor		
3:50	B		Sleeping		
3:55	M		Chatting with neighbor		
3:55	B		Sleeping		
4:00	B		Sleeping		
4:05	B		Sleeping		
4:10	M		Starting fire to boil water		
4:15	M		Waiting for water to boil		
4:20	M		Mixing guinea corn flour in a bowl with water to make a semi-solid paste	Guinea con flour, water	
4:25	M		Adding the guinea corn paste into boiling water and stirring		
4:30	M		Kunu is ready to be served	Kunun dawa	
4:30	M		Checking on sleeping baby		
4:35	B		Still sleeping		
4:40	M		Sweeping the surrounding		
4:40	M		sweeping		
4:45	M		Sweeping		
4:50	M		Washing the utensil		
4:55	M		Washing the utensils		
5:00	B		Child wakes up crying		
5:05	M	B	Carrying baby and consoling him		
5:10	M	B	Giving the child water	Water	
5:15	M	B	Bathing the child		
5:20	M	B	Bathing the child		
5:25	M	B	Dressing the baby		
5:30	M		Serving kunu from flask	Kunu dawa	
5:35	M		Adding MNP into a portion of kunu in a smaller cup and mixing it well ensuring it dissolves	Kunu dawa	
5:36	M	B	Feeding the child with a spoon		

TIME	ACTOR INITIALS	RECIPIENT INITIALS	WHAT HAPPENED	FOOD	Reactivity
5:36	B		Taking kunu from the mother with enthusiasm	Kunu dawa	
5:37	M	B	Feeding the child kunu	Kunu dawa	
5:38	M	B	feeding the child kunu	Kunu dawa	
5:39	M	B	Feeding the child kunu	Kunu dawa	
5:40	M	B	Feeding the child kunu with spoon. The baby finished	Kunun dawa	
5:40	M	B	Washing the child hands and mouth		
5:45	M	B	Carrying child on her back		
5:50	M	B,N1,N2	Chatting with neighbor in front of the house while carrying baby on the back		
5:55	M	B,N1,N2	Chatting with neighbor		
6:00	M	B	Carrying baby on her back sitting on stool in the house		

TIME	ACTOR INITIALS	RECIPIENT INITIALS	WHAT HAPPENED	FOOD	Reactivity

PARTICIPANT ID: __thatch house with zinc door _______

Primary Questions (Complete <u>after</u> you complete data collection):

From what you have seen observing THIS HOUSEHOLD, please answer the following questions in detail:

Question	Write your <u>detailed</u> response here
1. In this household, what would be the best way(s) to give nutritional supplements such as an MNP to young children to ensure compliance?<br><br>Why?	MNP can be given through the mother. The father is also an influence because he is charge of decisions in the household .<br><br>He asked the mother repeated if she had given the child MNP<br><br>Train the mother on MNP use and importance to ensure compliance.
2. In this household, who is favored during meal preparation or feeding?<br><br>How is this favoritism expressed?	The parents and older child are favored during meal preparation. The meal prepared was in a solid state.<br><br>The father and older child were favored because the quantity of their food was more.<br>The boys got a separate bowl<br>The husband had a separate bowl too<br>The baby was fed by the mother
3. How could we promote the MNP supplement for this family that would attract their attention?<br><br>Who would they listen to?	MNP can be promoted to this household through the mother and the father. The father has the final say in everything in the house because the woman admitted to wanting to stop administering. MNP when her child started purging but the husband told her continue and she did,<br><br>They will also listen to the community leader because the woman was chatting with neighbors on how they respect the community and listen to him. So also the religious leaders in the community.

4. In this household, what would be the best ways to promote the MNP but limit how much it would be shared?<br><br>Why?	The best way to limit MNP sharing in this household is to ensure that the mother understands that is meant for her 18 months old child only.<br>Even though she did not taste it and the older children were not around when she administered it. It can be emphasized during enlightenment.
5. What other issues and concerns did you see with this family that are important for us to consider with regard to introduction of an MNP?<br><br>Why?	The woman was lamenting on lack of adequate food given to the children. They do not always have access to all the ingredient to make rich kunu for their children. Ingredients like groundnut, soya beans and the rest. There was good hygiene in the house. She cleaned utensils immediately after use, washed hands and encouraged the children to do so too.<br><br>The child did not have a separate bowl when they were eating.
6. Based on your experience observing this child today, do you believe reactivity negatively impacted your ability to see the food-related behaviors of the child?<br><br>Please explain your answer in detail.	Reactivity did not affect the feeding behavior in this household. The mother said that is how the child usually eats and she is always responsible for feeding the child. It is only in cases where she step out that her husband feeds the child.
